# Randomized pilot study to disseminate caries-control services in dentist offices

**DOI:** 10.1186/1472-6831-6-7

**Published:** 2006-05-03

**Authors:** David Grembowski, Charles Spiekerman, Michael A del Aguila, Maxwell Anderson, Debra Reynolds, Allison Ellersick, James Foster, Leslie Choate

**Affiliations:** 1 Department of Dental Public Health Sciences, Box 357475, University of Washington, Seattle, WA, 98195, USA; 2 Department of Health Services, University of Washington, Box 357660, Seattle, WA, 98195, USA; 3 Delta Dental Data & Analysis Center, 9706 4^th ^Ave NE, Seattle, WA, 98115, USA; 4 Delta Dental Washington Dental Service, P.O. Box 75688, Seattle, WA, 98175-0688, USA

## Abstract

**Background:**

To determine whether education and financial incentives increased dentists' delivery of fluoride varnish and sealants to at risk children covered by capitation dental insurance in Washington state (U.S.).

**Methods:**

In 1999, 53 dental offices in Washington Dental Service's capitation dental plan were invited to participate in the study, and consenting offices were randomized to intervention (n = 9) and control (n = 10) groups. Offices recruited 689 capitation children aged 6–14 and at risk for caries, who were followed for 2 years. Intervention offices received provider education and fee-for-service reimbursement for delivering fluoride varnish and sealants. Insurance records were used to calculate office service rates for fluoride, sealants, and restorations. Parents completed mail surveys after follow-up to measure their children's dental utilization, dental satisfaction, dental fear and oral health status. Regression models estimated differences in service rates between intervention and control offices, and compared survey measures between groups.

**Results:**

Nineteen offices (34%) consented to participate in the study. Fluoride and sealant rates were greater in the intervention offices than the control offices, but the differences were not statistically significant. Restoration rates were lower in the intervention offices than the control offices. Parents in the intervention group reported their children had less dental fear than control group parents.

**Conclusion:**

Due to low dentist participation the study lacked power to detect an intervention effect on dentists' delivery of caries-control services. The intervention may have reduced children's dental fear.

## Background

Scientific advances and new, effective caries-control services have emerged for preventing caries, [1,2] yet most general dentists have not adopted them[3]. Dissemination, or efforts to persuade dentists to adopt effective innovations, is important for improving public health, particularly among children. Although caries has declined in the U.S. over the past three decades, 51% of children aged 5–9 and 78% of 17 year-olds have at least one carious lesion or filling [4-6]. Reliance on diffusion, or the passive spread of new technology, will not solve the problem, given evidence that application of medical technology lags an average of 17 years[7].

One approach to increase dentist adoption of new, caries-control services is to pay dentists for providing them. However, when fluoride varnish became a covered benefit in a fee-for-service dental plan in 1996, most dentists did not adopt the technology, [8] suggesting that stronger interventions are necessary to increase dentist adoption of caries-control services.

The purpose of this study is to determine whether a more intensive intervention composed of provider education and reimbursement for a package of caries-control services increases the delivery of caries-control services and reduces restorations among at risk children with capitation dental coverage.

### Caries Prevention Study

The Caries Prevention Study was conducted in the capitation dental plan offered by Washington Dental Service (WDS), the Delta Dental Plan in Washington state. As in most capitation plans, dentists receive a fixed, monthly payment for each capitation enrollee in the practice, and dentists provide all the care that may be required, within contract limitations, without additional payment. Thus, the dentist has a financial incentive – that is, earns more income – to prevent disease and avoid treatment costs. The plan fully covers fluoride varnish and sealants for children aged 14 and under. About 52% of U.S. children aged 6–18 with private dental insurance see a dentist each year[9].

The intervention has two parts: 1) fee-for-service (FFS) reimbursement for providing fluoride varnish and sealants to at risk children with capitation dental benefits; and 2) provider education about caries-control technologies and how to incorporate them into daily practice. The intervention promotes dentists' adoption by supplementing the dentist's capitation payment with fee-for-service reimbursement for sealants and fluoride varnish delivered to capitation children who are at risk for caries. Provider education included didactic instruction on caries-control services and the "business case" for dental practice based on prevention, plus a video demonstration of fluoride varnish application.

Economic theory and diffusion theory suggest the intervention will increase dentist adoption of sealants and fluoride varnish. Dentists have two financial incentives to adopt the technologies. Because financial reimbursement for caries-control services increases dentist income and dentists usually want to increase practice revenue, the financial incentive may increase dentist adoption of the technologies[10]. In addition, by delivering caries-control services and preventing decay, [11-17] restorations may be reduced, and therefore, dentists retain more profits from their capitation payments, an added incentive to adopt the technology.

Diffusion theory posits that innovations do not sell themselves but are adopted over time through the predictable patterns of communication in a profession[18,19]. Compared to reparative treatments, preventive innovations tend to have a slower rate of adoption because clinicians have difficulty observing their relative advantages[18]. The combination of provider education and financial reimbursement can speed-up the diffusion process because they increase provider awareness and knowledge of the innovation and its relative advantages[18]. In one study, dentists who knew more about fluoride varnish were more likely to adopt the technology than those who knew less[3]. Well-designed classes also create communication channels for sharing information that can promote adoption[20].

Adoption also is more likely to occur when interventions target the entire dental office rather than just the dentist, mainly because innovations almost always require changes in office structure and ways of working together[21]. Consequently, provider education about caries-control services included dental office staff as well as dentists. Finally, when outside professional dental organizations, such as WDS, sponsor the intervention and thereby set practice norms for caries-control services, organizations may increase the spread of innovations, although few studies exist on this topic[21].

In contrast, Kuhn's model of paradigm shift in scientific disciplines suggests the intervention will not increase dentist adoption of caries control services[22]. The intervention is more than the adoption of a new technology; it is a paradigm shift from the traditional surgical approach to a disease-based approach, or "medical model," of dental practice. For dentists, this requires a philosophical switch that can impose a significant change in the way clinicians provide care and generate income[1,3,23].

According to Kuhn, paradigm shift for a provider is not gradual; it is all-or-nothing. For the profession as a whole, paradigm shift can be a long-term process that begins when dentists realize that the old paradigm (the surgical model) no longer adequately addresses the problems facing the profession, and it progresses as more and more evidence supporting the new paradigm (the medical model) appears in the literature. Sufficient evidence exists presently for resin based sealants, but insufficient evidence exists for ionomor sealants[24]. Without an evidence base for most caries-control services, paradigm shift to the medical model is premature, and the intervention may not be strong enough to speed up this process.

In particular, dental offices may not adopt fluoride varnish because the service is not integrated fully into U.S. dental institutions. After the Federal Drug Administration approved fluoride varnish as a devise for use in 1994, it can be used "off-label," which means the agent is being used for another purpose for which FDA approval is lacking. Off-label use of fluoride varnish for caries prevention is occurring because of substantial evidence that fluoride varnish reduces caries[14]. The American Dental Association does not have a preventive procedure code for fluoride varnish, and all dental insurance plans do not reimburse for fluoride varnish, which may have slowed adoption of the service.

The evidence in medical and dental care is equivocal about whether financial incentives increase the delivery of preventive services [25-31]. Some studies report that incentives motivated medical providers to improve chart documentation but did not increase preventive services[25]. Even if financial incentives are effective, greater use of preventive services may not reduce restorations[32,33]. In contrast, small group interactive education, along with educational outreach by experts, are effective in changing provider behavior, particularly for preventive care[34].

Our purpose is to conduct a randomized study testing whether financial incentives and provider education increase dentists' delivery of caries-control services to children at risk of caries.

## Methods

### Study design, populations and intervention

The impact of the Caries Prevention Study on the delivery of dental services was evaluated using a randomized posttest-only control group study design[35]. In 1999 we invited 53 dentists who owned Seattle-area dental offices and were network providers in WDS's capitation dental plan, and who had 30 or more children patients aged 6–14 covered by the plan. In 1997 about 65% of capitation dentists provided dental sealants to at least 1 child, but among those dentists, only about 6% of their children patients received sealants. Of these children, an average of 3.1 sealants were provided per child. Accurate records of dentists' delivery of fluoride varnish did not exist before the study, and we assumed dentist delivery of fluoride varnish was similar to other dentists in Washington state[3,8]. In short, dentists had not fully adopted these two services at baseline. The study was reviewed and approved by the Institutional Review Board of the University of Washington.

About 36% of the dentist-owners (n = 19) consented to participate. The offices were randomized in a stratified manner to ensure the groups were reasonably balanced in terms of office size. The offices of the consenting dentist owners were ordered by number of children aged 6–14 in the office. In each successive pair of offices, one office was randomly chosen for the intervention group, and the other office was assigned to the control group, yielding 9 dentist-owners in the intervention group and 10 dentist-owners in the control group.

Intervention dentists and their office staff attended an educational session about caries-control services. Dentists received continuing dental education credits, and staff attended because Washington state law allows delegation of caries-control services to auxiliaries. The 4-hour didactic sessions provided information about the clinical benefits of fluoride varnish and sealants, how to incorporate them into day-to-day practice, and how the delivery of preventive services might contribute to the financial health of the practice. A video was shown demonstrating the application of fluoride varnish, and protocols for enrolling and following children were reviewed. Control dentists and staff attended separate training sessions covering study data collection protocols.

Between August 1999 and July 2000, intervention and control dental offices invited eligible children to participate in the study at regular office visits. Eligibility criteria were: 1) coverage by WDS capitation plan; 2) aged 6–14; 3) parental consent to participate; and 4) at risk for caries (defined as ≥ 1 restoration or carious lesion)[36]. WDS monitored utilization records to verify that all children with restorations seen at intervention and control officers were invited to participate in the study. A total of 391 intervention and 298 control children were enrolled.

Children were followed for 2 years through July 2002. Intervention dentists received their contractual capitation payments and monthly fee-for-service reimbursement for providing fluoride varnish ($20 U.S.) and sealants ($20 U.S.) to eligible children. To preserve the value of the incentive, intervention offices received free supplies of Duraphat^® ^fluoride varnish throughout the follow-up period. Intervention and control offices were required contractually to submit service records to WDS, and intervention offices received fee-for-service reimbursement when service records contained procedure codes for fluoride varnish (WDS code 1206), topical fluoride application (1201, 1203, 1204) or sealants (1351). Fluoride codes in WDS service records were compared with fluoride codes in office dental charts for sampled intervention and control children (n = 289). At the end of follow-up, intervention and control offices reported on the percentage of eligible children receiving fluoride varnish.

WDS mailed parents 6-month recall letters to promote regular visits. When children exited the study, WDS mailed parents a questionnaire about their children's oral health status, dental utilization and dental fear, and satisfaction with their child's dental care.

### Measures

#### Dental service rates

WDS service records were used to calculate dentist service rates, or the average number of times that a dental service was provided to enrolled children in the office of each dentist-owner in the 2-year follow-up period. Dental services rates were calculated for fluoride, sealants, and restorations.

#### Office characteristics

The characteristics of intervention and control offices included the number of capitation patients covered by WDS in the office, number of capitation children aged 6 to 14, and number of hygienists employed by the office.

#### Baseline caries risk

Dental offices recorded the number of decayed, missing, and filled teeth (dft and DFT) and the number of sealants for each child at enrollment. A child's caries risk at baseline was measured by summing the dft and DFT scores. Offices also reported their perception of a child's caries risk (low, moderate, or high), and whether the child was taking fluoride supplements, whether orthodontic treatment was underway, and brushing frequency.

#### Oral health status

Survey measures included the parent's self-rating of the child's oral health on a 5-point scale (poor (1), fair, good, very good, excellent (5). Parents also rated, compared to one year ago, the condition of the child's teeth on a 5-point scale (much better (1) to much worse (5).

#### Dental satisfaction and utilization

Parents rated their satisfaction with their children's dental care at the follow-up survey through two items: 1) parent rating of the dental care from the child's dentist; and 2) parent rating of the preventive services from the child's dentist. Each item was rated on a 5-point scale (poor (1), fair, good, very good, excellent (5)). Survey measures included the parent's self-report of whether the child received any sealants, fluoride varnish, or any restorations in the past year.

#### Dental fear

Parents rated their child's dental fear at the follow-up survey using a modified item from the Corah Dental Anxiety Scale[37]. Parents were asked how their child would feel if the child had to go to the dentist tomorrow. Children were categorized as being fearful if the parent responded the child would be afraid that it would be unpleasant or painful, or the child would be very frightened of what the dentist might do.

#### Child and household characteristics

Though the dental office, rather than the child, was the unit of randomization, child and parent characteristics might also influence dental utilization, dental satisfaction, and dental fear[38]. Child characteristics measured from WDS records included age, gender and years enrolled in the study, which may be less than two years for children of parents losing dental capitation benefits (range: 0–2 years). Child characteristics measured from the parent survey included use of fluoride drops or tablets and frequency of snacks, pop or juice between meals. Parent and household characteristics included age, gender, the parent's race/ethnicity, years of education, the parent's marital status, and the number of people in the household.

### Data collection

The office measures of a child's caries risk were collected using a "Tooth Chart," a version of the form previously field-tested and used in a statewide oral health survey of Washington children[39]. Protocols for completing the Tooth Charts were explained at training sessions. Offices completed Tooth Charts for each child at enrollment and each dental visit in the follow-up period. Intervention and control offices were reimbursed $10 (U.S.) by WDS for each completed Tooth Chart to increase compliance with study protocols and offset their data collection costs. Tooth Charts were reviewed for completeness, and offices were contacted to supply any missing data from office records when available.

The parent survey was performed by WDS and followed procedures recommended by Dillman[40,41]. When children exited the study, parents were mailed a questionnaire, cover letter, prepaid return envelope, and a $15 (U.S.) gift certificate. The initial mailing was followed by: (1) a reminder postcard; (2) a second mailing of the questionnaire and revised cover letter to nonrespondents; and (3) a third mailing of the questionnaire and revised cover letter to nonrespondents.

### Data analysis

Bivariate statistical tests compared the characteristics of participating and nonparticipating dentists and offices. Bivariate statistical tests were performed to determine whether the characteristics of children and dental offices in the intervention group were significantly different than the characteristics in the control group.

Treatment and control group differences in amounts of fluorides, sealants, and restorations given per child over the study period were evaluated using permutation tests for group-randomized data[42,43]. The permutation test makes no distributional assumptions, and as applied here, takes into account the stratified group randomization by using all randomizations possible via this scheme to create the permutation comparison distribution. The group differences were adjusted using linear modeling, taking into account the following covariates: time in study with dental capitation coverage, child characteristics (age, gender, current orthodontia, sum of dft and DFT, dentist-evaluated caries risk, and number of sealed teeth at study entry, fluoride supplement use in restoration model), size of the office's capitation plan (number of children and number of total patients in capitation plan), and the number of hygienists employed by the office. All *p*-values are two-sided. Computations were performed using *S-Plus*^© ^2000 statistical software[44].

Logistic regression was performed to determine whether the age and gender of children with completed questionnaires were different from those without, controlling for group. For those with questionnaires, bivariate statistical tests were performed to determine whether children, parents and households in the intervention group were significantly different from the control group. Regression models were estimated to determine intervention effects on parent-reported dental utilization, dental satisfaction, and dental fear, and all models employed the permutation tests to determine intervention effects. Children control variables (female, age, brushing, fluoride supplements, snacks) and adult/household control variables (adult education, nonwhite race, age, female, marital status, and household size) were included in the models.

## Results

### Participating and non-participating dentists

Dentists who chose to participate had significantly more children aged 6–14 years old in the capitation dental plan in the previous year than dentists who did not participate (avg 220 vs. 81 children, *p*=.004), and participating dentists had more total patients in the capitation plan (avg 1171 vs. 479, *p*=.007). Otherwise no statistically significant differences existed between participating and nonparticipating dentists at baseline for the following characteristics: percentage solo practitioners (75% vs. 72%, respectively); average number of hygienists (1.06 vs. .69); average number of assistants (3.04 vs. 2.67); average number of operatories (4.92 vs. 4.31); electronic submission of dental claims (46% vs. 34%); computer in office (79% vs. 86%); accepting new patients (92% vs. 90%); average percent of children with sealants based on WDS records (10% vs. 15%). The main reasons for nonparticipation were office disruption (31% of nonparticipating dentists), lack of interest (28%), and uncertain future membership in the capitation dental plan (21%); other concerns were office staff reluctance (10%) and the poor timing of the study for the office (10%).

### Children and dental office characteristics

Table 1 presents the characteristics of children and dental offices in the intervention and control groups. Although no statistically significant differences were found between groups, children in the intervention group tended to have more sealed teeth, a greater percentage of dentists rating their caries risk as high, and less likely to have orthodontia. Dental offices in the intervention group also tended to have more capitation patients and employ more hygienists.

**Table 1:** Baseline characteristics of dental offices

	**Control Group**	**Intervention Group**	***p*-value**
Dental Office Characteristics	(n = 10)	(n = 9)	
Average number of capitation adults and children aged 6–14 and adults	1042 (960)	1408 (1758)	.57
Average number of capitation children aged 6–14	196 (191)	270 (324)	.44
Average number of hygienists	0.8 (1.1)	1.7 (1.3)	.27

Standard deviations are in parentheses.

Of all the intervention and control offices, only two intervention offices submitted service records with the WDS procedure code (1206) specific for fluoride varnish, as opposed to codes for other fluoride applications. About 4% of all fluoride codes in chart records were for fluoride varnish. In interviews with office personnel after the follow-up period, about half of the offices in each group reported consistent use of fluoride varnish for most eligible children.

Table 3 presents unadjusted dental service rates for dental offices in the control and intervention groups. Overall, the average fluoride (either fluoride varnish or topical fluoride application) and sealant rates were greater in the intervention offices than in the control offices. Average restoration rates were similar in the intervention and control offices.

**Table 2:** Baseline characteristics of enrolled children

	**Control Group**	**Intervention Group**	***p*-value**
Child Characteristics	(n = 298)	(n = 391)	
Average length of follow-up	1.8 (0.5)	1.9 (0.4)	.83
Gender (% male)	55	53	.72
Average age (yrs)	10.0 (2.2)	9.9 (2.3)	.73
Average number of sealed teeth	1.0 (1.6)	1.9 (2.2)	.67
Dentist-evaluated caries risk (%)			.31
Low	13	12	
Medium	32	11	
High	9	30	
No evaluation	45	46	
Children with orthodontic treatment (%)	15	8	.61
Average number of decayed and filled teeth (sum of dft, DFT)	4.2 (2.7)	4.2 (2.7)	.99

Standard deviations are in parentheses.

**Table 3:** Unadjusted dental service rates for dental offices in the control and intervention groups

**Control Group**	**Number of Children**	**Average Number Services per Child in 2-Year Follow-up Period**		
**Offices**	**Followed**	**Fluoride**	**Sealants**	**Restorations**

1	2	0.5 (0.7)	0.0 (0.0)	0.0 (0.0)
2	5	2.6 (0.9)	0.0 (0.0)	0.6 (0.9)
3	7	2.3 (1.7)	0.1 (0.4)	0.4 (0.5)
4	10	1.7 (1.1)	0.7 (1.9)	0.6 (1.1)
5	21	2.8 (0.9)	1.2 (1.9)	1.8 (2.5)
6	21	0.9 (1.1)	0.9 (2.1)	1.7 (2.0)
7	27	1.1 (1.0)	0.9 (1.8)	2.9 (2.4)
8	28	2.4 (1.4)	1.3 (1.8)	1.1 (1.4)
9	74	3.0 (1.3)	0.9 (1.8)	1.9 (2.2)
10	103	1.8 (1.0)	0.6 (1.7)	2.5 (3.3)
**Total Children and Average Rates for All Control Offices**	298	2.1 (1.3)	0.8 (1.8)	2.0 (2.6)
				
**Intervention Group Offices**				
1	1	4.0 (-)	0.0 (-)	2.0 (-)
2	8	2.3 (1.2)	1.4 (1.7)	1.5 (1.1)
3	9	3.3 (1.2)	0.4 (1.3)	1.7 (2.1)
4	14	2.0 (1.2)	1.3 (1.4)	0.6 (1.2)
5	18	1.1 (1.0)	0.8 (1.6)	1.9 (2.2)
6	20	2.9 (1.2)	0.2 (0.7)	1.6 (1.6)
7	33	1.8 (0.9)	0.8 (1.6)	1.8 (2.4)
8	82	2.4 (1.2)	1.1 (1.7)	1.5 (2.2)
9	206	2.8 (1.3)	1.9 (2.3)	2.3 (2.6)
**Total Children and Average Rates for All Intervention Offices**	391	2.5 (1.3)	1.4 (2.1)	1.9 (2.4)

Standard deviations are in parentheses.

### Intervention effects on dental service rates

Table 4 presents adjusted differences in the dental service rates between the intervention and control offices. In the fluoride regression model, the fluoride rate was 0.19 greater in intervention than control offices, ninety-five percent confidence interval (-.30, 0.79). The estimated difference in the sealant rate between the intervention and control offices was 0.10 per child, 95% confidence interval (-.29, .41). Restoration rates were significantly lower, -0.46, in the intervention offices than in the control offices, 95% confidence interval (-.88, .00), *p*=.05.

**Table 4:** Adjusted differences in dental service rates between intervention and control dental offices

**Dental Service**	**Adjusted Difference in Rates (Intervention – Control)**	**95% confidence interval**	*p*-value*
Number of fluoride applications per child	0.19	(-.30, 0.79)	0.46
Number of sealants applied per child	0.10	(-.29, .41)	0.50
Number of restorations performed per child	-0.46	(-.88, .00)	0.05

* Control variables include time in study with dental capitation coverage, child characteristics (age, gender, current orthodontia, sum of dft and DFT, dentist-evaluated risk, number of sealed teeth at study entry, fluoride supplement use in restoration model), size of the office's capitation plan (number of children and number of total patients in capitation plan), and the number of hygienists employed by the office.

About 45% of the children were enrolled in two offices in the intervention and control groups, which might be contributing more to Table 4 results than the other offices. As a sensitivity analysis, when the two practices were excluded from regression models, similar results were obtained, but the restoration rates were no longer significant. When dentist-evaluated risk was excluded from the models because of a high percentage of missing values, the intervention effect on restorations was no longer significant.

### Parent survey

The parent follow-up survey had a 70% (n= 492) response rate. The children of parents who did or did not respond had similar age and gender (p > .05). Among survey respondents, the characteristics of children and parents were generally similar in the intervention and control groups (see Table 5). However, the intervention group had a higher percentage of female parents and smaller households than the control group.

**Table 5:** Characteristics of children, parents and households inthe follow-up parent survey

**Variable**	**Intervention Group (n = 282)**	**Control Group (n = 205)**	***p*-value**
Children Characteristics			
Percent female	47	45	.30
Average age (years)	10 (2.4)	10 (2.3)	.50
Percent brushing 2+ times daily	53	57	.48
Percent taking fluoride tablets or drops at home	9	11	.34
Percent eating snacks or drinking pop/juice 2+ times daily	64	59	.28
			
Respondent And Household Characteristics			
Percent female	80	70	.02
Average age (years)	40 (6.4)	40 (6.7)	.61
Average education (years)	14 (1.9)	14 (2.3)	.79
Percent nonwhite	9	15	.27
Percent single	15	14	.86
Household size	4.5 (1.5)	4.7 (1.4)	.04

Standard deviations are in parentheses.

Based on parents' reports, Table 6 presents group differences in children's dental utilization in the past 12 months. A greater percentage of intervention children received fluoride varnish and sealants, but these differences were not significant in regression models. No significant differences in restorations were reported. Parents in the intervention group also reported their children had less dental fear than control children.

**Table 6:** Group differences in dental utilization, satisfaction with child's dental care, child's dental fear and oral health status in the follow-up parent survey

**Variable**	**Intervention Group (n = 282)**	**Control Group (n = 205)**	*p*-value*
Dental Utilization			
Percentage children receiving:			
Fluoride varnish	65	53	.98
Sealants	33	27	.63
Any restorations	41	46	.69
			
Satisfaction With Dental Care			
Average satisfaction with dental care (5=excellent)	4.1 (0.9)	3.7 (1.0)	.17
Average satisfaction with preventive care (5=excellent)	4.2 (0.9)	3.7 (1.0)	.10
			
Dental Fear			
Average dental fear score (5=very fearful)	2.1 (0.7)	2.3 (0.9)	.04
			
Oral Health Status			
Average rating of child dental health now (5=excellent)	3.8 (0.9)	3.7 (0.9)	.37
Average rating of condition of teeth now compared to 1 year ago (1=much better)	2.3 (0.8)	2.5 (0.8)	.08

Standard deviations are in parentheses.

* Statistical tests adjust for children characteristics (gender, age, brushing, fluoride supplements, snacks) and adult/household characteristics (adult education, nonwhite race, age, gender, marital status, and household size).

## Discussion

We invited 53 dentists who owned their offices and were in Washington Dental Service's capitation dental plan to participate in the study. Less than half consented. Among consenting dentists, fluoride and sealant rates were greater in the intervention dental offices than the control offices, but the differences between the two groups were not statistically significant, likely because of small sample sizes from low dentist participation and therefore, lower numbers of participating children. Similar findings were obtained in the parent survey. In addition, intervention (and control) dental offices rarely used the WDS procedure code for fluoride varnish in WDS service records and office charts, indicating incomplete adoption of the service (offices typically used the American Dental Association's Current Dental Terminology code 1203 for child topical application of fluoride)[45]. Only half of the intervention offices self-reported using fluoride varnish consistently for eligible children.

We estimated intervention effects on fluoride rates, rather than fluoride varnish, because we lacked information distinguishing whether children received fluoride varnishes or topical fluoride applications. This occurred partly because incentive payments were not contingent on accurate coding, although incentives did improve chart documentation in some medical studies[25]. Offices may not have documented fluoride varnish because no preventive procedure code exists for the service in the American Dental Association's Current Dental Terminology – an indicator that fluoride varnish is not integrated fully into the profession[45]. If the intervention actually increased fluoride varnish, detecting this effect is harder when fluoride is the outcome measure, and, therefore, our results are conservative. In future studies, monitoring office coding might increase use of the procedure code for fluoride varnish, but monitoring also may increase the delivery of fluoride varnish in control offices.

We examined dentist and office characteristics associated with participation in the study. The number of capitation children in dental offices was the strongest predictor of office participation[46]. Dental offices with a smaller number of capitation children were less likely to participate, probably because participation promised small financial rewards. This result suggests that if future studies recruit offices with large numbers of at risk children, office participation rates may be high.

We may have found small differences between groups because the financial incentive lacked sufficient "economic clout" to cause increased delivery of caries-control services in intervention offices. Most intervention offices enrolled less than 50 at risk children, and total financial incentives paid to those offices were likely a very small percentage of annual revenues. This argument also suggests that financial incentives might be more effective in offices with a substantial number of at risk children.

A related factor is that dental offices had patients with both fee-for-service and capitation dental plans. When the majority of patients in an office has fee-for-service dental plans, the financial incentives of the fee-for-service plan may "spillover" and may affect how a dentist treats patients in capitation plans [47-49]. Potential spillover effects from fee-for-service dental plans may have diluted the effects of an intervention targeting capitation patients, providing additional justification for recruiting offices with large majorities of capitation patients in future studies.

The small differences between the intervention and control groups also may be caused by the Hawthorne effect. Medical studies indicate that physician behavior can be altered simply by the awareness their behavior is being monitored[50,51]. Control offices were monitored closely by WDS in the follow-up period to track recall patterns of enrolled children, and control offices performed oral health assessments at the office visits of enrolled children and submitted this information to WDS. Control offices may have knowingly or unknowingly increased the provision of caries control services as a result of observation (WDS monitoring) and adherence to data collection protocols, which may have reduced the differences between groups.

Controlling for child and office characteristics, restoration rates were lower in the intervention group than the control group. However, when dentist-evaluated risk was excluded from the models because of a high percentage of missing values, the intervention effect on restorations was no longer significant. For the offices reporting caries risk, control and intervention offices classified a similar percentage of children at low caries risk (13% vs. 12%, respectively). Control offices classified a higher percentage of children at medium risk than high risk (32% vs 9%), while intervention offices had an opposite pattern (11% medium risk vs. 30% high risk). Thus, a difference exists only at the medium-to-high threshold, which may reflect differences in how the dentists evaluated caries risk as well as differences in the oral health status of the children. Percentages might change if the caries risk data were complete. In short, restoration rates also may depend on dentists' clinical decisions and other factors, and the intervention effect may be contingent on controlling statistically for dentists' assessment of children's caries risk in the restoration regression model[32,52].

Overall, the pattern of significant and nonsignificant results in Table 4 indicates that intervention offices provided more caries control services and less restorative services than control offices, which may justify replication of the study with a larger sample of dental offices and children. In a prior study, Lennon et al, report that compared to fee-for-service payment, capitation payment was associated with greater preventive services and less restorations for children[53]. Thus, a central question of future studies is whether payment schemes that blend fee-for-service reimbursement with capitation coverage produce a similar, beneficial mix of preventive and restorative services among children patients.

Parents in the intervention group reported their children had less dental fear, were more satisfied with their children's preventive care, and were more likely to report improvements in their children's oral health, although the satisfaction and oral health effects were weak. Dental fear may be less in the intervention group because provider education may have improved skills in delivering noninvasive preventive services, which may have reduced fear among intervention children. Because higher dental prices are associated with higher quality of dental care, [54] the financial incentives may have increased the quality of preventive services in intervention offices, which may have increased parent satisfaction with preventive care. Parent perceptions of improved oral health, along with the other beneficial intervention effects, provide support for replicating the study in larger children populations.

If a future study with larger sample sizes and adequate power does not find statistically significant intervention effects, the results would be consistent with Kuhn's model explaining paradigm shifts in scientific disciplines[22]. His model predicts that paradigm shift from the traditional surgical approach to a disease-based approach of dental practice is a long-term process. Stronger and more comprehensive, multi-pronged interventions appear essential for overcoming the inertia of paradigm shift and speeding up dentist adoption of caries-control technologies[34,55,56]. Dissemination efforts targeting a specific caries-control service may be ineffective when the real issue is increasing paradigm shift.

Our findings are limited to Seattle-area dentists in the provider network of Washington Dental Service's capitation dental plan and who consented to participate in the study. In addition, findings are limited to Seattle-area children aged 6–14 who were at risk for caries, covered by the capitation dental plan, and seen by participating dentists. A limitation of the study is inadequate sample sizes to detect small intervention effects. Greater financial incentives or different provider education may have different results.

## Conclusion

Because dentists with greater numbers of capitation children were more likely to participate, we recommend that future studies increase sample sizes by recruiting dental offices which have a majority of patients in capitation dental plans, which also may reduce potential spillover effects. Findings suggest the intervention may have reduced children's dental fear.

## Competing interests

The Caries Prevention Study was a collaboration between the University of Washington and Washington Dental Service (the Delta Dental insurance plan in Washington state), and the majority of funds for the study, including the financial incentives paid to dentists, were provided by Washington Dental Service. Given findings that the intervention did not increase the delivery of caries-control services to at risk children, any potential conflict of interest for the co-authors at Washington Dental Service may not be an issue.

## Authors' contributions

All authors read and approved the final manuscript.

David Grembowski: principal investigator responsible for the conduct of the overall study and first author

Charles Spiekerman: biostatistician responsible for randomization of dentist offices, performance of data analyses, preparing data analysis and results sections of manuscript

Michael A. del Aguila: lead investigator at Washington Dental Service, with overall responsibility for overseeing the implementation of the study in the dental offices

Maxwell Anderson: vice president of Washington Dental Service who influenced the design of the study, secured WDS resources for the study, designed and participated in the provider education component of the intervention, and assisted in the interpretation of results

Debra Reynolds: lead field representative responsible for the day-to-day conduct of the study in the dental offices

Allison Ellersick: lead data manager responsible for managing day-to-day collection of study data, including the conduct of the mail survey of parents

James Foster: responsible for constructing the study's data base at Washington Dental Service

Leslie Choate: responsible for supporting all phases of data collection at Washington Dental Service

## Pre-publication history

The pre-publication history for this paper can be accessed here:


